# Crystal structure of 4,6-dimethyl-2-[(2,3,4,6-tetra-*O*-acetyl-β-d-galacto­pyranos­yl)sulfan­yl]pyrimidine

**DOI:** 10.1107/S205698901901449X

**Published:** 2019-11-05

**Authors:** Mamdouh A. Abu-Zaied, Galal H. Elgemeie, Peter G. Jones

**Affiliations:** a Green Chemistry Department, National Research Centre, 33 El Bohouth Street, Dokki, Giza, Egypt; b Chemistry Department, Faculty of Science, Helwan University, Cairo, Egypt; c Institut für Anorganische und Analytische Chemie, Technische Universität Braunschweig, Hagenring 30, D-38106 Braunschweig, Germany

**Keywords:** crystal structure, galactose, pyrimidine, weak hydrogen bond

## Abstract

In the title com­pound, C_20_H_26_N_2_O_9_S, the S atom is attached equatorially to the sugar ring. The C—S bond lengths are unequal, with S—C_s_ = 1.8018 (13) Å and S—C_p_ = 1.7662 (13) Å (s = sugar and p = pyrimid­yl). In the crystal, a system of three weak hydrogen bonds, sharing an oxygen acceptor, links the mol­ecules to form chains propagating parallel to the *b*-axis direction.

## Abstract 2

In the title com­pound, the S atom is attached equatorially to the sugar ring. The C—S bond lengths are unequal. In the crystal, a system of three weak hydrogen bonds, sharing an oxygen acceptor, links the mol­ecules to form chains propagating parallel to the *b*-axis direction.

## Chemical context   

Nucleosides are building blocks of biological systems and display a wide range of biological activities (Ding *et al.*, 2003 ▸). Pyrimidine nucleoside analogues provide diverse and novel moieties for pharmacological targets, and they play basic and com­prehensive roles in the field of medicinal chemistry (Xu *et al.*, 2017 ▸). Different strategies for the synthesis of many pyrimidine nucleoside analogues have been developed to access new and potent pharmacological agents (Cao *et al.*, 2011 ▸). Many such derivatives are manufactured as potential chemotherapeutic agents and have a significant impact on current medicinal research (Ohkubo *et al.*, 2012 ▸). Recently, thio­glycosides have proved to be important in the production of medically important carbohydrate com­pounds, because of their ease of preparation and chemical stability (Gourdain *et al.*, 2011 ▸).

We have recently described the preparation of various pyrimidine and pyridine thio­glycosides that displayed antagonistic activity (Hammad *et al.*, 2018 ▸; Elgemeie *et al.*, 2010 ▸). We have also reported the use of di­hydro­pyridine thio­glycosides as substrates or inhibitors of protein glycosyl­ation (Scale *et al.*, 1997 ▸; Elgemeie *et al.*, 2015 ▸, 2016 ▸, 2017 ▸) and the use of pyrimidine thio­glycosides as anti­hepatocellular carcinoma agents (Elgemeie & Farag, 2017 ▸). Continuing our efforts to develop simple and cost-effective methodologies for the synthesis of pyrimidine thio­glycosides, we report here the one-step synthesis of a pyrimidine-2-thio­galactoside derivative by the reaction of 4,6-di­methyl­pyrimidine-2(1*H*)-thione (**1**) with 2,3,4,6-tetra-*O*-acetyl-α-d-galactopyranosyl bromide (**2**). This reaction in NaH/DMF at room temperature gave a product for which two isomeric structures seemed possible, corresponding to two possible modes of glysosylation to give the pyrimidine-*N*-galactoside (**3**) or its regioisomer pyrimidine-2-thio­galac­to­side **4** (see Scheme). Spectroscopic data cannot differentiate between these structures. It has been suggested that **1** reacts with **2** *via* a simple S_N_2 reaction to give the β-glycoside product **4** (Davis, 2000 ▸).

## Structural commentary   

The crystal structure determination indicated unambiguously the formation of the pyrimidine-2-thio­galactoside, **4**, as the only product in the solid state.
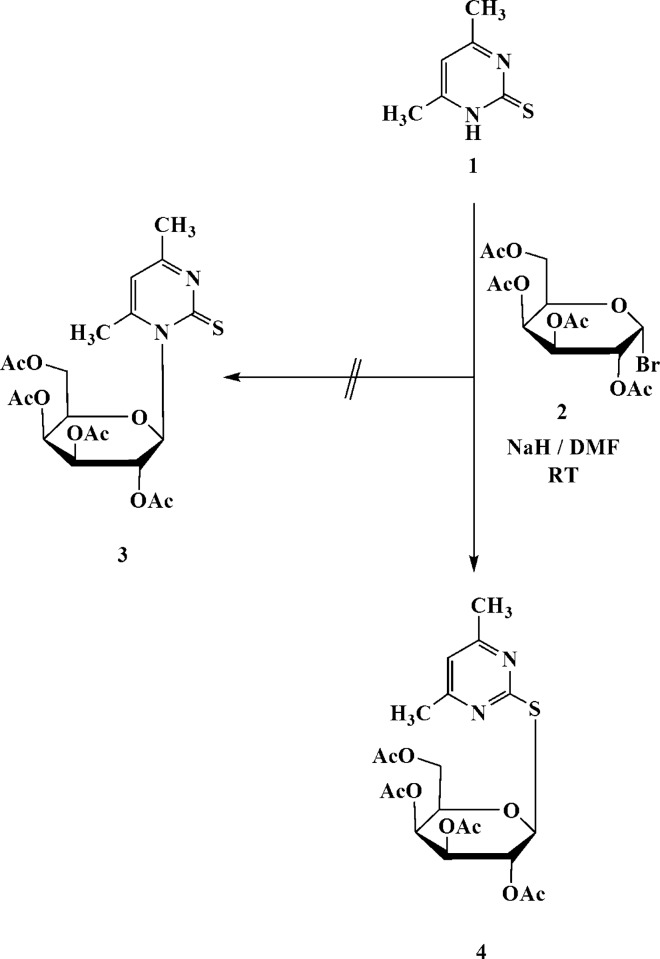


**Figure 1:**
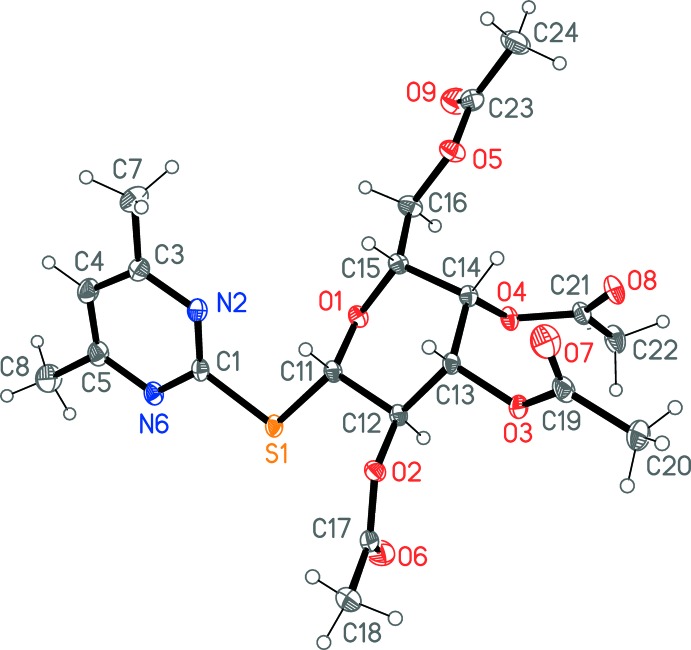
The mol­ecular structure of the title com­pound, **4**, in the crystal. Displacement ellipsoids represent 50% probability levels.

The mol­ecular structure of **4** is shown in Fig. 1 ▸ (for selected torsion angles, see Table 1 ▸) and the S atom is attached equatorially to the sugar ring. Similar to the structure of a related glucose derivative (Masoud *et al.*, 2017 ▸), the C—S bond lengths are unequal, with S—C_s_ = 1.8018 (13) Å and S—C_p_ = 1.7662 (13) Å (s = sugar and p = pyrimid­yl). The relative orientation of the pyridyl ring and the sugar moiety is defined by the torsion angles N2—C1—S1—C11 [−7.85 (12)°] and C1—S1—C11—C12 [165.01 (9)°]. All the acetyl groups show extended conformations, with absolute C—O—C—C torsion angles in the range 173–179°.

## Supra­molecular features   

**Figure 2:**
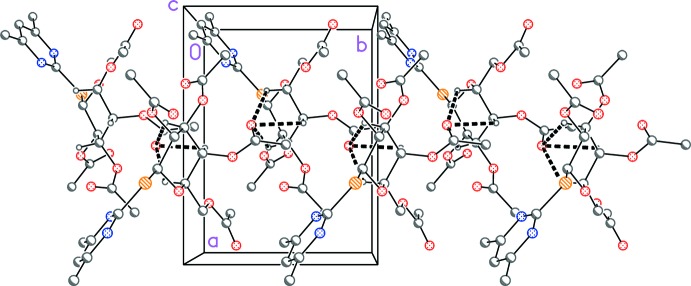
Packing diagram of **4** projected parallel to the *ab* plane in the region *z* ≃ 1. Dashed lines indicate weak C—H⋯O hydrogen bonds. H atoms not involved in this hydrogen bonding system have been omitted.

Some short C—H⋯O and C—H⋯S contacts are listed in Table 2 ▸, but these are at best borderline ‘weak’ hydrogen bonds, particularly in view of their narrow angles. The mol­ecular packing is thus rather featureless. However, a motif of three sugar-ring C—H groups (C13—H13, C14—H14 and C15—H15) sharing a common acceptor (O8) can be recognized (Fig. 2 ▸). Neighbouring mol­ecules are connected *via* the 2_1_ operator, leading to chains of mol­ecules propagating parallel to the *b*-axis direction.

## Database survey   

A search of the Cambridge Structural Database (Vwersion 2.0.0; Groom *et al.*, 2016 ▸) for tetra­acetyl thio­glycosides with an S-bonded heterocycle [linkage S—C(—N)_2_, restricted to hexoses] gave one hit, a 1,2,4-triazole derivative of tetra­acetyl­glucose (refcode HEKPUL; El Ashry *et al.*, 2018 ▸).

## Synthesis and crystallization   

To a solution of pyrimidine-2(1*H*)-thione (**1**; 1.40 g, 0.01 mol) in dry DMF (20 ml), NaH (15 mmol) was added gradually over a period of 15 min and the solution was stirred at room temperature for another 30 min. A solution of 2,3,4,6-tetra-*O*-acetyl-α-d-galacto­pyranosyl bromide (**2**; 4.52 g, 0.011 mol) in DMF (20 ml) was then added dropwise over a period of 30 min and the reaction mixture was stirred at room temperature until the reaction was judged com­plete by thin-layer chromatography (3–6 h). The mixture was evaporated under reduced pressure at 333 K and the residue was washed with distilled water to remove potassium bromide. The crude solid was collected by filtration and purified using column chromatography (the solvent system was petroleum ether/ethyl acetate, 3:1 *v*/*v*; *R*_F_ = 0.35); after evaporation of the solvent, this afforded com­pound **4** as colourless crystals in 85% yield (m.p. 441.2 K). IR (KBr, cm^−1^): ν 1752 (C=O); ^1^H NMR (500 MHz, DMSO-*d*_6_): δ 2.11 (*s*, 12H, 4 × OAc), 2.45 (*s*, 6H, 2CH_3_), 4.01–4.12 (*m*, 2H, 2H-6′), 4.35–4.37 (*m*, 1H, H-5′), 5.21 (*t*, 1H, *J*_4′-3′_ = 2.6, *J*_4′-5′_ = 2.4 Hz, H-4′), 5.42–5.46 (*m*, 2H, H-3′, H-2′), 5.98 (*d*, 1H, *J*_1′-2′_ = 10.65 Hz, H-1′), 7.01 (*s*, 1H, pyrimidine H-5); ^13^C NMR: δ 21.43 (4 × OAc), 22.4 (2CH_3_), 62.13 (C-6′), 68.41 (C-5′), 71.12 (C-4′), 74.43 (C-3′), 77.56 (C-2′), 82.12 (C-1′), 118.41 (C-5), 168.35 (C-4), 170.45 (C-6), 172.78 (4 × C=O). Analysis calculated (%) for C_20_H_26_N_2_O_9_S: C 51.06, H 5.57, N 5.95, S 6.82; found: C 51.16, H 5.46, N 5.82, S 6.75.

## Refinement   

Crystal data, data collection and structure refinement details are summarized in Table 3 ▸. Methyl groups were refined as idealized rigid groups allowed to rotate but not tip (C—H = 0.98 Å and H—C—H = 109.5°). Other H atoms were included using a riding model starting from calculated positions (aromatic C—H = 0.95 Å, methyl­ene C—H = 0.99 Å and methine C—H = 1.00 Å).

## Supplementary Material

Crystal structure: contains datablock(s) I, global. DOI: 10.1107/S205698901901449X/hb7861sup1.cif

Structure factors: contains datablock(s) I. DOI: 10.1107/S205698901901449X/hb7861Isup2.hkl

CCDC references: 1962352, 1962352

Additional supporting information:  crystallographic information; 3D view; checkCIF report

## Figures and Tables

**Table 1:** Selected torsion angles (°)

S1—C11—C12—C13	178.21 (9)	C22—C21—O4—C14	177.90 (11)
S1—C11—O1—C15	171.60 (8)	C24—C23—O5—C16	178.85 (13)
C18—C17—O2—C12	176.21 (11)	C15—C16—O5—C23	174.82 (12)
C20—C19—O3—C13	−173.83 (12)		

**Table 2:** Hydrogen-bond geometry (Å, °)

*D*—H⋯*A*	*D*—H	H⋯*A*	*D*⋯*A*	*D*—H⋯*A*
C7—H7*C*⋯O9^i^	0.98	2.57	3.495 (2)	157
C8—H8*B*⋯O1^ii^	0.98	2.52	3.2499 (18)	131
C13—H13⋯O8^iii^	1.00	2.65	3.2998 (16)	123
C14—H14⋯O8^iii^	1.00	2.53	3.0626 (16)	113
C15—H15⋯O8^iii^	1.00	2.50	3.1759 (16)	124
C18—H18*B*⋯S1^iv^	0.98	2.95	3.7876 (19)	144
C22—H22*C*⋯O6^v^	0.98	2.51	3.1911 (19)	127

Symmetry codes: (i) 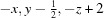; (ii) 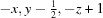; (iii) 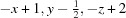; (iv) 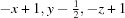; (v) 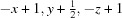.

**Table 3:** Experimental details

Crystal data	
Chemical formula	C_20_H_26_N_2_O_9_S
*M*_r_	470.49
Crystal system, space group	Monoclinic, *P*2_1_
Temperature (K)	100
*a*, *b*, *c* (Å)	11.4868 (2), 8.6444 (2), 11.5561 (2)
β (°)	91.3762 (16)
*V* (Å^3^)	1147.14 (4)
*Z*	2
Radiation type	Mo *K*α
μ (mm^−1^)	0.19
Crystal size (mm)	0.40 × 0.40 × 0.08

Data collection	
Diffractometer	Oxford Diffraction Xcalibur Eos
Absorption correction	Multi-scan (*CrysAlis PRO*; Rigaku OD, 2015 ▸)
*T*_min_, *T*_max_	0.896, 1.000
No. of measured, independent and observed [*I* > 2σ(*I*)] reflections	107162, 7825, 7530
*R*_int_	0.034
(sin θ/λ)_max_ (Å^−1^)	0.757

Refinement	
*R*[*F* ^2^ > 2σ(*F* ^2^)], *wR*(*F* ^2^), *S*	0.028, 0.073, 1.04
No. of reflections	7825
No. of parameters	295
No. of restraints	1
H-atom treatment	H-atom parameters constrained
Δρ_max_, Δρ_min_ (e Å^−3^)	0.34, −0.21
Absolute structure	Flack *x* determined using 3355 quotients [(*I* ^+^)−(*I* ^−^)]/[(*I* ^+^)+(*I* ^−^)] (Parsons *et al.*, 2013 ▸)
Absolute structure parameter	−0.003 (11)

Computer programs: *CrysAlis PRO* (Rigaku OD, 2015 ▸), *SHELXS97* (Sheldrick, 2008 ▸), *SHELXL2017* (Sheldrick, 2015 ▸) and *XP* (Siemens, 1994 ▸).

## supplementary crystallographic information

### Crystal data

**Table:**

C_20_H_26_N_2_O_9_S	*F*(000) = 496
*M**_r_* = 470.49	*D*_x_ = 1.362 Mg m^−^^3^
Monoclinic, *P*2_1_	Mo *K*α radiation, λ = 0.71073 Å
*a* = 11.4868 (2) Å	Cell parameters from 34705 reflections
*b* = 8.6444 (2) Å	θ = 2.5–31.9°
*c* = 11.5561 (2) Å	µ = 0.19 mm^−^^1^
β = 91.3762 (16)°	*T* = 100 K
*V* = 1147.14 (4) Å^3^	Plate, colourless
*Z* = 2	0.40 × 0.40 × 0.08 mm

### Data collection

**Table:**

Oxford Diffraction Xcalibur Eos diffractometer	7825 independent reflections
Radiation source: fine-focus sealed X-ray tube	7530 reflections with *I* > 2σ(*I*)
Detector resolution: 16.1419 pixels mm^-1^	*R*_int_ = 0.034
ω–scan	θ_max_ = 32.6°, θ_min_ = 2.5°
Absorption correction: multi-scan (CrysAlis PRO; Rigaku OD, 2015)	*h* = −16→17
*T*_min_ = 0.896, *T*_max_ = 1.000	*k* = −13→12
107162 measured reflections	*l* = −17→17

### Refinement

**Table:**

Refinement on *F*^2^	Secondary atom site location: difference Fourier map
Least-squares matrix: full	Hydrogen site location: inferred from neighbouring sites
*R*[*F*^2^ > 2σ(*F*^2^)] = 0.028	H-atom parameters constrained
*wR*(*F*^2^) = 0.073	*w* = 1/[σ^2^(*F*_o_^2^) + (0.0448*P*)^2^ + 0.1562*P*] where *P* = (*F*_o_^2^ + 2*F*_c_^2^)/3
*S* = 1.04	(Δ/σ)_max_ = 0.003
7825 reflections	Δρ_max_ = 0.34 e Å^−^^3^
295 parameters	Δρ_min_ = −0.21 e Å^−^^3^
1 restraint	Absolute structure: Flack *x* determined using 3355 quotients [(I+)-(I-)]/[(I+)+(I-)] (Parsons *et al.*, 2013)
Primary atom site location: structure-invariant direct methods	Absolute structure parameter: −0.003 (11)

### Special details

**Table:**

Geometry. All esds (except the esd in the dihedral angle between two l.s. planes) are estimated using the full covariance matrix. The cell esds are taken into account individually in the estimation of esds in distances, angles and torsion angles; correlations between esds in cell parameters are only used when they are defined by crystal symmetry. An approximate (isotropic) treatment of cell esds is used for estimating esds involving l.s. planes.

### Fractional atomic coordinates and isotropic or equivalent isotropic displacement parameters (Å^2^)

**Table:**

	*x*	*y*	*z*	*U*_iso_*/*U*_eq_	
S1	0.31717 (3)	0.37712 (4)	0.52391 (3)	0.01490 (7)	
C1	0.20586 (11)	0.24276 (15)	0.55326 (12)	0.0142 (2)	
N2	0.19449 (10)	0.18868 (14)	0.65991 (10)	0.0157 (2)	
C3	0.10603 (12)	0.08858 (17)	0.67531 (12)	0.0172 (2)	
C4	0.03201 (12)	0.04670 (18)	0.58372 (14)	0.0206 (3)	
H4	−0.030920	−0.022627	0.594791	0.025*	
C5	0.05295 (11)	0.10958 (19)	0.47507 (13)	0.0199 (3)	
N6	0.14080 (10)	0.20913 (15)	0.45864 (10)	0.0171 (2)	
C7	0.09350 (14)	0.0274 (2)	0.79576 (14)	0.0245 (3)	
H7A	0.168605	−0.013057	0.824151	0.037*	
H7B	0.035507	−0.055784	0.795217	0.037*	
H7C	0.068210	0.110957	0.846639	0.037*	
C8	−0.02150 (15)	0.0695 (3)	0.37087 (15)	0.0321 (4)	
H8A	−0.006740	0.143333	0.308590	0.048*	
H8B	−0.103788	0.074109	0.391103	0.048*	
H8C	−0.002582	−0.035284	0.344863	0.048*	
C11	0.36873 (11)	0.41461 (15)	0.66971 (11)	0.0131 (2)	
H11	0.375290	0.314664	0.712943	0.016*	
C12	0.48727 (11)	0.49574 (15)	0.67193 (11)	0.0128 (2)	
H12	0.483420	0.593888	0.626193	0.015*	
C13	0.52391 (10)	0.52856 (15)	0.79714 (11)	0.0126 (2)	
H13	0.543513	0.428968	0.837101	0.015*	
C14	0.42901 (11)	0.61209 (15)	0.86310 (10)	0.0125 (2)	
H14	0.450359	0.615925	0.947450	0.015*	
C15	0.31396 (11)	0.52721 (16)	0.84512 (11)	0.0134 (2)	
H15	0.320387	0.421966	0.880626	0.016*	
C16	0.21073 (12)	0.61091 (19)	0.89541 (11)	0.0182 (2)	
H16A	0.199582	0.712822	0.857662	0.022*	
H16B	0.138732	0.549329	0.883778	0.022*	
C17	0.60600 (13)	0.42066 (17)	0.51651 (12)	0.0189 (3)	
C18	0.70211 (15)	0.3144 (2)	0.48332 (15)	0.0268 (3)	
H18A	0.701248	0.301910	0.399020	0.040*	
H18B	0.691163	0.213438	0.520012	0.040*	
H18C	0.777016	0.358241	0.509150	0.040*	
C19	0.70586 (11)	0.60977 (19)	0.88007 (12)	0.0195 (3)	
C20	0.80074 (13)	0.7270 (2)	0.86755 (15)	0.0289 (3)	
H20A	0.777357	0.824463	0.903511	0.043*	
H20B	0.814694	0.744261	0.785231	0.043*	
H20C	0.872252	0.688885	0.905729	0.043*	
C21	0.47753 (11)	0.87850 (18)	0.87510 (11)	0.0161 (2)	
C22	0.46064 (15)	1.03358 (18)	0.81936 (13)	0.0229 (3)	
H22A	0.484169	1.114920	0.874164	0.034*	
H22B	0.378397	1.047057	0.797118	0.034*	
H22C	0.508294	1.040209	0.750322	0.034*	
C23	0.15859 (13)	0.71433 (19)	1.07533 (13)	0.0219 (3)	
C24	0.19319 (16)	0.7243 (3)	1.20142 (14)	0.0306 (4)	
H24A	0.135476	0.785614	1.242427	0.046*	
H24B	0.269721	0.773711	1.209540	0.046*	
H24C	0.196923	0.619929	1.234481	0.046*	
O1	0.28647 (8)	0.51166 (12)	0.72429 (8)	0.01402 (17)	
O2	0.57416 (8)	0.39412 (12)	0.62726 (8)	0.01502 (18)	
O3	0.62667 (8)	0.62251 (13)	0.79139 (8)	0.01642 (18)	
O4	0.41523 (8)	0.76733 (12)	0.81822 (8)	0.01450 (18)	
O5	0.23709 (9)	0.63008 (14)	1.01710 (9)	0.0198 (2)	
O6	0.56194 (12)	0.51912 (15)	0.45620 (10)	0.0278 (2)	
O7	0.69797 (10)	0.51640 (16)	0.95647 (10)	0.0266 (2)	
O8	0.53897 (9)	0.85198 (13)	0.95903 (9)	0.0201 (2)	
O9	0.07262 (10)	0.76925 (17)	1.03075 (11)	0.0295 (3)	

### Atomic displacement parameters (Å^2^)

**Table:**

	*U*^11^	*U*^22^	*U*^33^	*U*^12^	*U*^13^	*U*^23^
S1	0.01701 (13)	0.01501 (14)	0.01247 (12)	−0.00376 (11)	−0.00397 (9)	−0.00027 (11)
C1	0.0126 (5)	0.0123 (6)	0.0177 (5)	0.0001 (4)	−0.0024 (4)	−0.0023 (4)
N2	0.0156 (5)	0.0138 (5)	0.0176 (5)	0.0000 (4)	−0.0019 (4)	0.0000 (4)
C3	0.0151 (5)	0.0148 (6)	0.0218 (6)	0.0007 (4)	0.0013 (4)	0.0003 (5)
C4	0.0143 (5)	0.0210 (7)	0.0265 (7)	−0.0033 (5)	−0.0001 (5)	−0.0024 (5)
C5	0.0136 (5)	0.0224 (7)	0.0235 (6)	−0.0022 (5)	−0.0030 (5)	−0.0043 (5)
N6	0.0144 (5)	0.0191 (6)	0.0177 (5)	−0.0013 (4)	−0.0037 (4)	−0.0033 (4)
C7	0.0223 (6)	0.0257 (8)	0.0257 (7)	−0.0023 (6)	0.0014 (5)	0.0072 (6)
C8	0.0232 (7)	0.0468 (11)	0.0260 (7)	−0.0135 (7)	−0.0066 (6)	−0.0073 (7)
C11	0.0139 (5)	0.0129 (6)	0.0123 (5)	−0.0003 (4)	−0.0036 (4)	−0.0004 (4)
C12	0.0139 (5)	0.0126 (6)	0.0120 (5)	−0.0005 (4)	−0.0026 (4)	−0.0009 (4)
C13	0.0118 (5)	0.0136 (6)	0.0122 (5)	−0.0016 (4)	−0.0029 (4)	−0.0008 (4)
C14	0.0142 (5)	0.0118 (5)	0.0114 (5)	0.0002 (4)	−0.0031 (4)	0.0011 (4)
C15	0.0139 (5)	0.0149 (6)	0.0112 (5)	0.0009 (4)	−0.0027 (4)	0.0009 (4)
C16	0.0159 (5)	0.0242 (7)	0.0142 (5)	0.0030 (5)	−0.0017 (4)	−0.0005 (5)
C17	0.0222 (6)	0.0199 (7)	0.0148 (5)	−0.0085 (5)	0.0028 (4)	−0.0051 (5)
C18	0.0255 (7)	0.0288 (8)	0.0266 (7)	−0.0049 (6)	0.0080 (6)	−0.0112 (6)
C19	0.0132 (5)	0.0263 (7)	0.0188 (6)	0.0007 (5)	−0.0036 (4)	−0.0079 (5)
C20	0.0169 (6)	0.0392 (10)	0.0303 (8)	−0.0094 (6)	−0.0020 (5)	−0.0098 (7)
C21	0.0202 (5)	0.0137 (6)	0.0142 (5)	−0.0009 (5)	−0.0014 (4)	−0.0028 (5)
C22	0.0356 (8)	0.0137 (6)	0.0189 (6)	−0.0028 (6)	−0.0062 (5)	0.0010 (5)
C23	0.0187 (6)	0.0250 (7)	0.0221 (6)	−0.0031 (5)	0.0056 (5)	−0.0031 (5)
C24	0.0286 (7)	0.0445 (11)	0.0189 (7)	−0.0026 (7)	0.0045 (6)	−0.0080 (7)
O1	0.0138 (4)	0.0156 (4)	0.0125 (4)	0.0015 (3)	−0.0042 (3)	−0.0007 (3)
O2	0.0156 (4)	0.0163 (5)	0.0131 (4)	−0.0001 (3)	0.0001 (3)	−0.0018 (3)
O3	0.0141 (4)	0.0201 (5)	0.0149 (4)	−0.0046 (4)	−0.0029 (3)	−0.0025 (4)
O4	0.0195 (4)	0.0108 (4)	0.0129 (4)	−0.0004 (3)	−0.0055 (3)	0.0005 (3)
O5	0.0183 (4)	0.0270 (6)	0.0142 (4)	0.0031 (4)	−0.0005 (3)	−0.0020 (4)
O6	0.0396 (6)	0.0273 (6)	0.0167 (5)	−0.0032 (5)	0.0034 (4)	0.0030 (4)
O7	0.0231 (5)	0.0330 (7)	0.0233 (5)	0.0017 (5)	−0.0102 (4)	0.0011 (5)
O8	0.0243 (5)	0.0184 (5)	0.0173 (4)	0.0006 (4)	−0.0076 (4)	−0.0046 (4)
O9	0.0207 (5)	0.0371 (7)	0.0310 (6)	0.0060 (5)	0.0038 (4)	−0.0013 (5)

### Geometric parameters (Å, º)

**Table:**

S1—C1	1.7662 (13)	C23—O9	1.201 (2)
S1—C11	1.8018 (13)	C23—O5	1.3511 (18)
C1—N2	1.3275 (18)	C23—C24	1.504 (2)
C1—N6	1.3414 (17)	C4—H4	0.9500
N2—C3	1.3497 (18)	C7—H7A	0.9800
C3—C4	1.390 (2)	C7—H7B	0.9800
C3—C7	1.499 (2)	C7—H7C	0.9800
C4—C5	1.394 (2)	C8—H8A	0.9800
C5—N6	1.3432 (18)	C8—H8B	0.9800
C5—C8	1.501 (2)	C8—H8C	0.9800
C11—O1	1.4223 (15)	C11—H11	1.0000
C11—C12	1.5314 (17)	C12—H12	1.0000
C12—O2	1.4349 (16)	C13—H13	1.0000
C12—C13	1.5239 (17)	C14—H14	1.0000
C13—O3	1.4356 (15)	C15—H15	1.0000
C13—C14	1.5267 (18)	C16—H16A	0.9900
C14—O4	1.4460 (16)	C16—H16B	0.9900
C14—C15	1.5214 (17)	C18—H18A	0.9800
C15—O1	1.4305 (15)	C18—H18B	0.9800
C15—C16	1.5168 (19)	C18—H18C	0.9800
C16—O5	1.4409 (16)	C20—H20A	0.9800
C17—O6	1.204 (2)	C20—H20B	0.9800
C17—O2	1.3591 (16)	C20—H20C	0.9800
C17—C18	1.493 (2)	C22—H22A	0.9800
C19—O7	1.201 (2)	C22—H22B	0.9800
C19—O3	1.3583 (16)	C22—H22C	0.9800
C19—C20	1.498 (2)	C24—H24A	0.9800
C21—O8	1.2077 (16)	C24—H24B	0.9800
C21—O4	1.3584 (16)	C24—H24C	0.9800
C21—C22	1.498 (2)		
			
C1—S1—C11	99.32 (6)	H7A—C7—H7C	109.5
N2—C1—N6	127.96 (13)	H7B—C7—H7C	109.5
N2—C1—S1	119.85 (10)	C5—C8—H8A	109.5
N6—C1—S1	112.19 (10)	C5—C8—H8B	109.5
C1—N2—C3	116.07 (12)	H8A—C8—H8B	109.5
N2—C3—C4	121.02 (13)	C5—C8—H8C	109.5
N2—C3—C7	115.98 (13)	H8A—C8—H8C	109.5
C4—C3—C7	123.00 (13)	H8B—C8—H8C	109.5
C3—C4—C5	117.95 (13)	O1—C11—H11	109.3
N6—C5—C4	121.56 (13)	C12—C11—H11	109.3
N6—C5—C8	116.76 (14)	S1—C11—H11	109.3
C4—C5—C8	121.68 (14)	O2—C12—H12	110.6
C1—N6—C5	115.43 (12)	C13—C12—H12	110.6
O1—C11—C12	108.80 (10)	C11—C12—H12	110.6
O1—C11—S1	108.29 (8)	O3—C13—H13	109.4
C12—C11—S1	111.73 (9)	C12—C13—H13	109.4
O2—C12—C13	106.07 (10)	C14—C13—H13	109.4
O2—C12—C11	109.85 (10)	O4—C14—H14	109.9
C13—C12—C11	109.05 (10)	C15—C14—H14	109.9
O3—C13—C12	105.66 (10)	C13—C14—H14	109.9
O3—C13—C14	110.69 (10)	O1—C15—H15	109.1
C12—C13—C14	112.20 (10)	C16—C15—H15	109.1
O4—C14—C15	108.14 (10)	C14—C15—H15	109.1
O4—C14—C13	109.47 (10)	O5—C16—H16A	110.5
C15—C14—C13	109.39 (10)	C15—C16—H16A	110.5
O1—C15—C16	105.22 (10)	O5—C16—H16B	110.5
O1—C15—C14	110.44 (10)	C15—C16—H16B	110.5
C16—C15—C14	113.71 (11)	H16A—C16—H16B	108.7
O5—C16—C15	106.32 (10)	C17—C18—H18A	109.5
O6—C17—O2	123.07 (14)	C17—C18—H18B	109.5
O6—C17—C18	126.10 (14)	H18A—C18—H18B	109.5
O2—C17—C18	110.82 (13)	C17—C18—H18C	109.5
O7—C19—O3	123.23 (13)	H18A—C18—H18C	109.5
O7—C19—C20	126.41 (14)	H18B—C18—H18C	109.5
O3—C19—C20	110.37 (13)	C19—C20—H20A	109.5
O8—C21—O4	123.04 (13)	C19—C20—H20B	109.5
O8—C21—C22	125.63 (13)	H20A—C20—H20B	109.5
O4—C21—C22	111.33 (11)	C19—C20—H20C	109.5
O9—C23—O5	123.46 (14)	H20A—C20—H20C	109.5
O9—C23—C24	126.07 (15)	H20B—C20—H20C	109.5
O5—C23—C24	110.45 (14)	C21—C22—H22A	109.5
C11—O1—C15	110.79 (9)	C21—C22—H22B	109.5
C17—O2—C12	116.15 (11)	H22A—C22—H22B	109.5
C19—O3—C13	117.11 (11)	C21—C22—H22C	109.5
C21—O4—C14	115.54 (10)	H22A—C22—H22C	109.5
C23—O5—C16	114.90 (11)	H22B—C22—H22C	109.5
C3—C4—H4	121.0	C23—C24—H24A	109.5
C5—C4—H4	121.0	C23—C24—H24B	109.5
C3—C7—H7A	109.5	H24A—C24—H24B	109.5
C3—C7—H7B	109.5	C23—C24—H24C	109.5
H7A—C7—H7B	109.5	H24A—C24—H24C	109.5
C3—C7—H7C	109.5	H24B—C24—H24C	109.5
			
C11—S1—C1—N2	−7.85 (12)	C12—C13—C14—C15	49.77 (14)
C11—S1—C1—N6	171.73 (10)	O4—C14—C15—O1	63.93 (13)
N6—C1—N2—C3	−0.6 (2)	C13—C14—C15—O1	−55.22 (14)
S1—C1—N2—C3	178.90 (10)	O4—C14—C15—C16	−54.09 (13)
C1—N2—C3—C4	−0.2 (2)	C13—C14—C15—C16	−173.24 (11)
C1—N2—C3—C7	179.87 (13)	O1—C15—C16—O5	−179.18 (11)
N2—C3—C4—C5	0.9 (2)	C14—C15—C16—O5	−58.19 (14)
C7—C3—C4—C5	−179.23 (15)	C12—C11—O1—C15	−66.76 (12)
C3—C4—C5—N6	−0.8 (2)	S1—C11—O1—C15	171.60 (8)
C3—C4—C5—C8	179.22 (15)	C16—C15—O1—C11	−171.37 (11)
N2—C1—N6—C5	0.7 (2)	C14—C15—O1—C11	65.52 (13)
S1—C1—N6—C5	−178.84 (10)	O6—C17—O2—C12	−2.78 (19)
C4—C5—N6—C1	0.1 (2)	C18—C17—O2—C12	176.21 (11)
C8—C5—N6—C1	−179.96 (14)	C13—C12—O2—C17	−140.59 (11)
C1—S1—C11—O1	−75.17 (9)	C11—C12—O2—C17	101.70 (12)
C1—S1—C11—C12	165.01 (9)	O7—C19—O3—C13	6.0 (2)
O1—C11—C12—O2	174.55 (9)	C20—C19—O3—C13	−173.83 (12)
S1—C11—C12—O2	−65.93 (12)	C12—C13—O3—C19	−150.10 (11)
O1—C11—C12—C13	58.70 (13)	C14—C13—O3—C19	88.21 (13)
S1—C11—C12—C13	178.21 (9)	O8—C21—O4—C14	−1.39 (18)
O2—C12—C13—O3	69.45 (12)	C22—C21—O4—C14	177.90 (11)
C11—C12—C13—O3	−172.30 (10)	C15—C14—O4—C21	146.97 (11)
O2—C12—C13—C14	−169.84 (10)	C13—C14—O4—C21	−93.93 (12)
C11—C12—C13—C14	−51.59 (14)	O9—C23—O5—C16	0.4 (2)
O3—C13—C14—O4	49.20 (13)	C24—C23—O5—C16	178.85 (13)
C12—C13—C14—O4	−68.56 (13)	C15—C16—O5—C23	174.82 (12)
O3—C13—C14—C15	167.53 (10)		

### Hydrogen-bond geometry (Å, º)

**Table:**

*D*—H···*A*	*D*—H	H···*A*	*D*···*A*	*D*—H···*A*
C7—H7*C*···O9^i^	0.98	2.57	3.495 (2)	157
C8—H8*B*···O1^ii^	0.98	2.52	3.2499 (18)	131
C13—H13···O8^iii^	1.00	2.65	3.2998 (16)	123
C14—H14···O8^iii^	1.00	2.53	3.0626 (16)	113
C15—H15···O8^iii^	1.00	2.50	3.1759 (16)	124
C18—H18*B*···S1^iv^	0.98	2.95	3.7876 (19)	144
C22—H22*C*···O6^v^	0.98	2.51	3.1911 (19)	127

Symmetry codes: (i) −*x*, *y*−1/2, −*z*+2; (ii) −*x*, *y*−1/2, −*z*+1; (iii) −*x*+1, *y*−1/2, −*z*+2; (iv) −*x*+1, *y*−1/2, −*z*+1; (v) −*x*+1, *y*+1/2, −*z*+1.
